# LncRNA LEGLTBC Functions as a ceRNA to Antagonize the Effects of miR-34a on the Downregulation of SIRT1 in Glucolipotoxicity-Induced INS-1 Beta Cell Oxidative Stress and Apoptosis

**DOI:** 10.1155/2019/4010764

**Published:** 2019-10-14

**Authors:** Xiang Kong, Chong-xiao Liu, Guo-dong Wang, Hui Yang, Xin-ming Yao, Qiang Hua, Xiao-yong Li, Hong-mei Zhang, Ming-zhe Ma, Qing Su, Kun Lv

**Affiliations:** ^1^Key Laboratory of Noncoding RNA Transformation Research of Anhui Higher Education Institution (Wannan Medical College), Wuhu 241002, China; ^2^Department of Endocrinology, The First Affiliated Hospital of Wannan Medical College, Yijishan Hospital, Wuhu 241001, China; ^3^Central Laboratory of Yijishan Hospital, Wuhu 241001, China; ^4^Department of Endocrinology, Seventh People's Hospital of Shanghai University of Traditional Chinese Medicine, Shanghai 200137, China; ^5^Department of Endocrinology, Xinhua Hospital, Shanghai Jiaotong University School of Medicine, Shanghai 200092, China; ^6^Anhui Provincial Engineering Research Center for Polysaccharide Drugs, School of Pharmacy, Wannan Medical College, Wuhu 241002, China; ^7^Department of Gastric Surgery, Fudan University Shanghai Cancer Center, Shanghai 200032, China

## Abstract

Type 2 diabetes mellitus is a chronic metabolic disorder characterized by elevated blood glucose and/or high serum free fatty acids. Chronic hyperlipidemia causes the dysfunction of pancreatic beta cells, which is aggravated in the presence of hyperglycemia (glucolipotoxicity). Long noncoding RNAs (lncRNAs) have been suggested to play key roles in type 1 diabetes mellitus development. However, their roles in glucolipotoxicity-induced beta cell dysfunction are not fully understood. In the present study, we identified the differentially expressed lncRNAs in INS-1 cells exposed to high glucose and palmitate (HG/PA). Among the dysregulated lncRNAs, NONRATT003679.2 (low expression in glucolipotoxicity-treated beta cells (LEGLTBC)) was involved in glucolipotoxicity-evoked rat islet beta cell damage. LEGLTBC functioned as a molecular sponge of miR-34a in INS-1 cells. Additionally, SIRT1 was identified as a target of miR-34a and LEGLTBC promoted SIRT1 expression by sponging miR-34a. The upregulation of LEGLTBC attenuated HG/PA-induced INS-1 cell injury through the promotion of SIRT1-mediated suppression of ROS accumulation and apoptosis. This is the first study to comprehensively identify the lncRNA expression profiling of HG/PA-treated INS-1 beta cells and to demonstrate that LEGLTBC functions as a competing endogenous RNA and regulates miR-34a/SIRT1-mediated oxidative stress and apoptosis in INS-1 cells undergoing glucolipotoxicity.

## 1. Introduction

Abundant nutrient intake in combination with decreased exercise contributes to the global epidemic of obesity and type 2 diabetes mellitus (T2DM), which is characterized by insulin resistance and/or pancreatic beta cell dysfunction [1]. A common feature of T2DM individuals is elevated blood glucose and/or high serum free fatty acids (FFA) [2]. Chronic elevation of hyperglycemia causes beta cell failure characterized by impaired insulin secretion and enhanced islet beta cell apoptosis (glucotoxicity) [3, 4]. Furthermore, long-term exposure of beta cells to FFA triggers apoptosis (lipotoxicity) and the elevated glucose augments fatty acid-induced beta cell death (glucolipotoxicity) [5–7]. The glucolipotoxicity plays a pivotal role in the worsening of beta cell function over time, which has been implicated in the development of T2DM [8]. Nonetheless, the molecular mechanisms involved in glucolipotoxicity-induced beta cell dysfunction are not fully understood.

As yet, studies have focused mainly on protein-coding genes, giving minor attention to the majority of eukaryotic transcriptomes, noncoding RNAs (ncRNAs). In the context of T2DM, microRNAs (miRNAs) are the most studied ncRNAs [9, 10]. The miRNAs, such as miR-34a, miR-146, miR-199-5p, and miR-139-5p, play important roles in the control of islet beta cell differentiation, function, and mass during the development of T2DM [11–13]. In addition to miRNAs, the long noncoding RNAs (lncRNAs), defined as transcripts longer than 200 nucleotides, have been identified as another class of functional molecules. Recent studies have identified the expression profiles of lncRNAs in beta cells after exposure to a combination of proinflammatory cytokines using microarray analysis [14, 15]. Differentially expressed lncRNAs have been confirmed in islets of NOD mice, a well-known model of type 1 diabetes mellitus [15]. However, to the best of our knowledge, there are no data regarding the expression patterns, targets, and functions of lncRNAs in beta cells after exposure to high-glucose and palmitate- (HG/PA-) induced glucolipotoxicity.

For the first time, we here aim to identify the lncRNAs that were differentially expressed in HG/PA-treated INS-1 beta cells by RNA sequencing (RNA-Seq). Some of the interesting lncRNAs with considerable alterations were verified by quantitative real-time reverse transcription PCR (qRT-PCR) in INS-1 cells and rat pancreatic islets after HG/PA treatment. Among the dysregulated lncRNAs, NONRATT003679.2 was markedly downregulated (fold change: 5.49; *P* = 1.28 × 10^−8^) in HG/PA-treated INS-1 beta cells, and which for simplicity will be hereafter referred to as lncRNA LEGLTBC (low expression in glucolipotoxicity-treated beta cells). The effects of LEGLTBC on glucolipotoxicity-mediated INS-1 cell damage were further investigated. The present research might provide significant new insights into the pathogenesis of glucolipotoxicity-induced beta cell dysfunction.

## 2. Materials and Methods

### 2.1. Islet Isolation, Cell Culture, and Treatment

As described in our previous studies [16, 17], rat INS-1 beta cells (a gift from Prof. Xiao Wang, Shanghai Institute of Endocrine and Metabolic Diseases, passages 30-35) were grown in RPMI 1640 medium containing 11.1 mM glucose, 10% fetal bovine serum, 50 *μ*M 2-mercaptoethanol, 10 mM HEPES, 2 mM L-glutamine, 1 mM sodium pyruvate, 100 U/mL penicillin, and 100 *μ*g/mL streptomycin. All reagents were purchased from Invitrogen (Carlsbad, CA). As described in our previous report [16], PA was dissolved in 0.1 M sodium hydroxide to prepare a stock solution (100 mM). Five percent (wt/vol) fatty-acid-free BSA (Roche, USA) solution was prepared in double-distilled H_2_O. A 5 mM PA/5% BSA solution was prepared by complexing an appropriate amount of PA to 5% BSA. The above solution was then cooled to room temperature and diluted in RPMI 1640 to final concentrations.

INS-1 cells were grown to confluence, stimulated with 0.5 mM PA in the presence of 25 mM glucose (HG) for 24 h. The cell line and incubation conditions were chosen because 25 mM glucose/0.5 mM PA has been demonstrated to induce obvious glucolipotoxicity in INS-1 cells. Accordingly, this cell model has been used to explore the glucolipotoxicity-induced changes of mRNA microarray chips and proteomics [18, 19].

Isolated islets were obtained from 10-week-old male Sprague-Dawley (SD) rats (SLAC Laboratory Animal Co., Shanghai, China). All of the animals were maintained at the Experimental Animal Centre of Xinhua Hospital. All studies were approved by the Institutional Animal Care and Use Committee at the Xinhua Hospital, Shanghai Jiaotong University School of Medicine. Pancreatic islets were isolated by collagenase digestion (Collagenase P, Roche) followed by Ficoll gradient purification and used after repeated washes with Hanks' balanced salt solution. Rat islets were cultured in RPMI 1640 medium and stimulated with 25 mM glucose/0.5 mM palmitate for 48 h.

### 2.2. LncRNA Identification by RNA-Seq

TRIzol Reagent (Invitrogen, CA, USA) was used to extract total RNA from INS-1 cells. Total RNA was quantified using a NanoDrop ND-2000 spectrophotometer (Thermo Fisher Scientific, USA), and RNA integrity was determined using an Agilent 2100 Bioanalyzer (Agilent Technologies, USA). Strand-specific libraries were prepared using the VAHTS Total RNA-Seq Library Prep Kit for Illumina (Vazyme, China) following the manufacturer's instructions. Cluster was generated by cBot with the library diluted to 10 pM and then sequenced on the HiSeq 2500 System (Illumina, USA).

Sequencing raw reads were preprocessed by filtering out rRNA reads, sequencing adapters, short-fragment reads, and other low-quality reads using seqtk. HISAT2 (version: 2.0.4) was used to map the cleaned reads to the rat reference genome (Rnor_6.0) with two mismatches. After genome mapping, StringTie (version: 1.3.0) was run with a reference annotation to generate FPKM values (Fragments Per Kilobase of exon model per Million mapped reads) for known gene models. Differentially expressed genes were identified using edgeR.

StringTie (version: 1.3.0) was used to assemble reads into transcripts. Putative lncRNAs were defined as novel transcripts set through the following filters: length ≥ 200 bp; number of exons ≥ 2.0; ORF ≤ 300 bp; and have no or weak protein-coding ability. Finally, we used edgeR to integrate the RNA-Seq-derived lncRNAs with the known lncRNAs previously annotated by NONCODE v4. The significant differentially expressed lncRNAs with the fold changes of the threshold values ≥ 2.0 or ≤−2.0 (*p* ≤ 0.05) were selected. Data are accessed via GSE124833.

### 2.3. Real-Time Quantitative RT-PCR (qRT-PCR) Analyses

A two-step reaction process, reverse transcription (RT) and PCR, was performed. Reaction cDNA was synthesized using the PrimeScript™ RT Master Mix (TAKARA, Japan). Real-time PCR was performed using the LightCycler® 480 II Real-Time PCR Instrument (Roche, Swiss). The expression levels of lncRNAs or miR-34a were normalized to *β*-actin or U6 and were calculated using the 2^-ΔΔCt^ method. The primer sequences were listed in Table 1.

### 2.4. Subcellular Fractionation Location

To determine the cellular localization of LEGLTBC, cytoplasmic and nuclear fractions of INS-1 cells were isolated and collected with the nuclear/cytoplasmic isolation kit (BioVision Inc., USA) according to the manufacturer's instructions. After that, total RNA were extracted from the collections of both the cytoplasm and the nucleus and cDNA was synthesized for the evaluation of LEGLTBC. RNAs extracted from each of the fractions were subjected to the following RT-PCR analysis of the levels of the nuclear control transcript (U6) and the cytoplasmic control transcript (*β*-actin).

### 2.5. Transfection

The designed siRNAs against LEGLTBC (si-LEGLTBC), silence information regulator 1 (SIRT1, si-SIRT1), and negative control (si-NC), as well as the miR-34a mimic (miR-34a), miRNA negative control (miR-NC), miR-34a inhibitor (anti-miR-34a), and miRNA inhibitor negative control (anti-NC) were purchased from RiboBio Co. (Guangzhou, China). The designed pcDNA 3.1-LEGLTBC (LEGLTBC) and pcDNA empty vector (vector) were purchased from HanHeng Trading Co. Ltd. (Shanghai, China). INS-1 cells or rat islets were seeded into 24-well or 96-well plates and treated for 48 h with the designed siRNA, miRNA inhibitor, or plasmids using the Lipofectamine 2000 transfection reagent (Thermo Fisher Scientific, USA) before stimulation with HG/PA.

### 2.6. Assessment of Apoptosis in INS-1 Cells

As described in our previous report [17], the INS-1 cells were stained with Annexin V-FITC and propidium iodide (PI) (BD Biosciences, CA, USA) and then subjected to flow cytometry to assess apoptosis. Apoptotic cells were shown as Annexin V-positive cells.

### 2.7. Detection of Intracellular ROS Generation in INS-1 Cells

As described in our previous studies [16, 17], intracellular ROS levels were detected using the 2′,7′-dichlorodihydrofluorescin diacetate (DCFH-DA, Beyotime Biotechnology Inc., China) probe through the fluorescence microscope or microplate reader. After treatment for the indicated time periods, the INS-1 cells or rat islets were washed with RPMI medium containing no fetal bovine serum and incubated with 10 *μ*M DCFH-DA for 30 min at 37°C. Then, the INS-1 cells or rat islets were washed twice with RPMI medium containing no fetal bovine serum, and the mean fluorescence intensity (MFI) of DCF was immediately measured through the Multi-Mode Microplate Reader (BioTek Instruments Inc., VT, USA; excitation 490 nm, emission 530 nm). The fluorescence of DCF was monitored with a fluorescence microscope (Leica Microsystems, Germany). The intracellular ROS levels were calculated using the MFI and calibrated with the total RNA concentration.

### 2.8. Luciferase Reporter Assay

The wild-type LEGLTBC (containing miR-34a binding sites) or mutated LEGLTBC sequence (mutant in miR-34a binding sites) was amplified using PCR and cloned into the pmirGLO Vector (Promega, USA) for luciferase reporter assay using the one-step directed cloning kit (Novoprotein, Shanghai, China). Primers used in this study are listed in Table 1. For luciferase assay, the INS-1 cells were plated in 24-well culture plates and then cotransfected with the reporter plasmids of pmirGLO-LEGLTBC-WT or the mutant derivative devoid of miR-34a binding sites (LEGLTBC-MUT) and miR-34a mimic or miR-NC. Luciferase activity was measured 48 h after transfection using the Dual-Luciferase Reporter Assay System (Promega, USA).

### 2.9. RNA Immunoprecipitation (RIP) Assay

RIP experiment was performed to verify the relationship between LEGLTBC and miR-34a using the EZ-Magna RIP Kit (Millipore, USA). Briefly, the INS-1 cells were lysed in complete RIP lysis buffer containing a protease inhibitor and an RNase inhibitor. Then, 100 *μ*L extract was incubated with argonaute 2 (AGO2) antibody or control immunoglobulin G (IgG; Millipore, USA) for 8 h at 4°C, followed by adding protein A/G beads. Next, the beads were incubated with proteinase K to digest the proteins. Finally, purified RNA was subjected to qRT-PCR analysis using specific primers for LEGLTBC.

### 2.10. Western Blot Analysis

Equal protein (40 *μ*g) of INS-1 cells or rat islets was separated by electrophoresis on a sodium dodecyl sulphate polyacrylamide gel (SDS-PAGE), electrotransferred to polyvinylidene fluoride membranes (Millipore, USA), and then incubated with the primary antibodies overnight and with the correspondent secondary antibodies. An antibody against SIRT1 was purchased from Cell Signaling Technology Inc. (MA, USA). Antibodies against cleaved caspase 3 and tubulin were purchased from Beyotime Biotechnology Inc. (Nantong, China). Blots were quantified by densitometry using ImageJ software [20]. The intensity of the bands was normalized to that of tubulin.

### 2.11. Statistical Analysis

Data were shown as the mean ± standard deviation (S.D.) derived from the indicated number of independent experiments given in the figure legends. All data conform to the normal distribution using the Shapiro-Wilk normality test. Equality of variances was determined by using an *F*-test or a Levene's test. The differences among groups were determined by the use of one-way analysis of variance followed by the Newman-Keuls test or the Games-Howell test, while Student's *t*-test or Welch's *t*-test was performed for comparisons between two groups. The relationship between LEGLTBC and miR-34a was analyzed using Spearman's correlation analysis. A *P* value of less than 0.05 was considered to be statistically significant.

## 3. Results

### 3.1. Gene Expression Analysis and Validation

**Figure 1:**
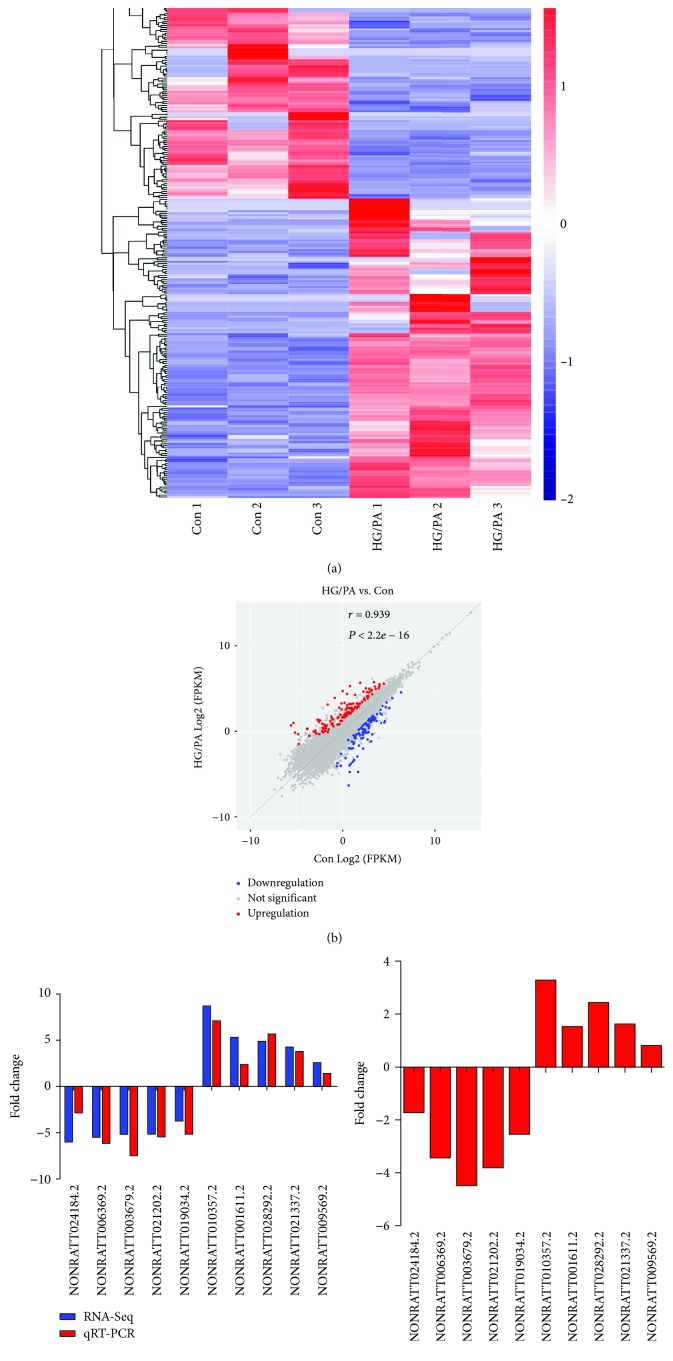
Profile of RNA-Seq data for lncRNAs. (a) Hierarchical clustering shows distinguished lncRNA expression profiling between control (Con) and HG/PA-treated INS-1 cells (3 replicates). Red, high expression; blue, low expression. (b) Scatter plot displays the variation of lncRNAs between Con and HG/PA-treated INS-1 cells. Red, upregulation; blue, downregulation. (c) The results reveal that the ten lncRNAs were downregulated or upregulated in HG/PA-treated INS-1 cells relative to control INS-1 cells. The qRT-PCR results were correlated well with the RNA-Seq data. (d) The results show the expression of these lncRNAs in HG/PA-treated rat islets compared to control islets. Data are the mean of ±SD of 5 independent experiments.

RNA-Seq analysis (biological triplicate) was performed to compare the control INS-1 cells with the HG/PA-treated INS-1 cells. According to the expression profiling data, 264 significantly dysregulated lncRNAs were identified with a set filter (fold change ≥ 2.0, *P* < 0.05) in the HG/PA-treated INS-1 cells: 153 were upregulated, while 111 were downregulated. Data are available via Gene Expression Omnibus (GEO) GSE124833. The total number of annotated lncRNAs is shown in [Supplementary-material supplementary-material-1]. The hierarchical clustering revealed the lncRNA expression patterns in different samples (Figure 1(a)). The variation of lncRNA expression was shown with a scatter plot (Figure 1(b)). A total of ten differentially expressed lncRNAs were randomly selected to validate the RNA-Seq data using qRT-PCR. The results of the qRT-PCR were largely consistent with the data from the RNA-Seq analysis (Figure 1(c)). In addition, similar results were observed in islets of rats incubated with the same HG/PA (Figure 1(d)).

### 3.2. GO Analysis

To further explore the potential functions of dysregulated lncRNAs induced by glucolipotoxicity, a functional enrichment analysis of the mRNAs coexpressed with these lncRNAs was employed. Based on our GO analysis ([Supplementary-material supplementary-material-1], [Supplementary-material supplementary-material-1]), the differentially expressed lncRNAs were mainly associated with the cell death signaling pathway (such as the regulation of the extrinsic apoptotic signaling pathway via death domain receptors (GO:1902043), the regulation of the release of cytochrome c from mitochondria (GO:0090200), the regulation of oxidative stress-induced cell death (GO:1903202), and the regulation of mitochondrial membrane permeability involved in the apoptotic process (GO:1902108)) and the intrinsic apoptotic signaling pathway in response to endoplasmic reticulum stress (GO:0070059).

### 3.3. LEGLTBC Is Involved in Glucolipotoxicity-Induced INS-1 Cell Apoptosis and ROS Generation

According to previous studies [15, 21], criteria were applied for the selection of the four candidate upregulated lncRNAs (Table 2), including high relative expression, available full sequence, and confidence in no protein-coding capacity. The evaluation of the potential coding capability of lncRNA NONRATT003679.2 was displayed in [Supplementary-material supplementary-material-1]. The results were as follows: although two short open reading frames (ORF 1 and 7) with more than 200 nt were predicted using the ORF Finder from the National Center for Biotechnology Information, none of their AUGs showed the Kozak consensus and no homologous protein sequences were found using a BLAST search. The transcript's noncoding nature was suggested by a negative score with CPC (coding potential calculator, the online protein-coding potential assessment software).

Knockdown of lncRNA NONRATT010357.2 or upregulation of lncRNA NONRATT003679.2 significantly reduced HG/PA-induced INS-1 cell death in our preliminary experiments, while inhibition of lncRNA NONRATT015294.2 or overexpression of lncRNA NONRATT024184.2 had no obvious effects (data not shown). Interestingly, only lncRNA NONRATT003679.2 was found to mediate HG/PA-induced reactive oxygen species (ROS) production. Therefore, lncRNA NONRATT003679.2 (spanning nearly 1128 base pairs) was selected for further analysis, which for simplicity will be hereafter referred to as lncRNA LEGLTBC (low expression in glucolipotoxicity-treated beta cells). Phylogenetic analysis using the UCSC Genome Browser (http://genome.ucsc.edu/) revealed that LEGLTBC is moderately conserved between humans and rats ([Supplementary-material supplementary-material-1]). As shown in [Supplementary-material supplementary-material-1], qRT-PCR was performed to detect the expression of LEGLBTC in SD rat tissues, including pancreatic, heart, liver, spleen, lung, muscle, and brain tissues, and in islets of rats. LEGLBTC appeared to be highly expressed in pancreatic tissues and islets of rats.

**Figure 2:**
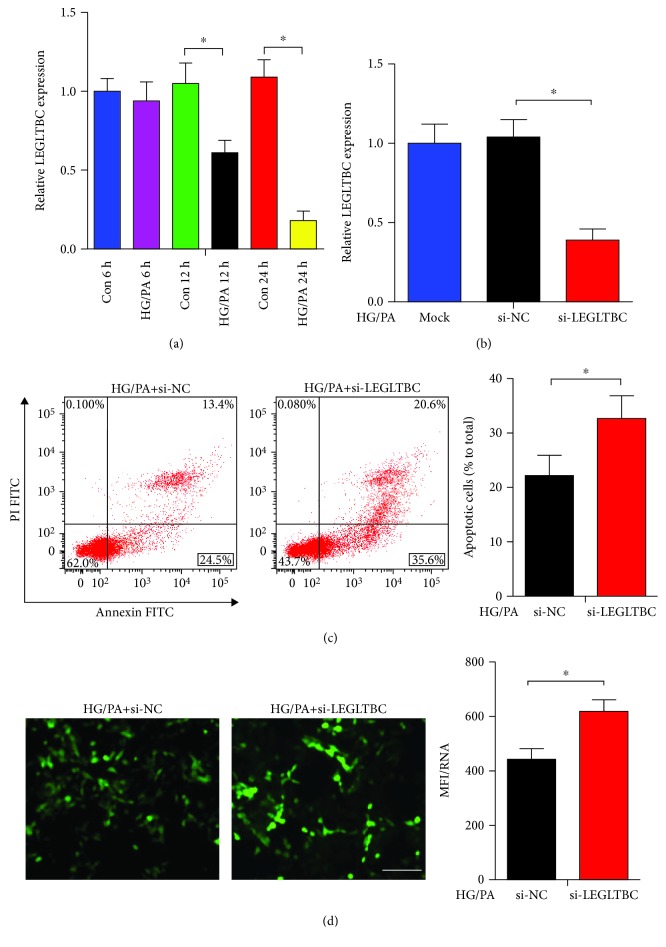
Silencing LEGLTBC induces oxidative stress and apoptosis in glucolipotoxicity. (a) The expression of LEGLTBC in HG/PA-treated INS-1 cells was assessed by qRT-PCR analysis. (b) Downregulation of LEGLTBC using siRNA for 48 h as detected by qRT-PCR analysis. (c) INS-1 cells were stained with Annexin V-FITC and PI and then subjected to flow cytometry to measure apoptosis. Apoptotic cells shown as Annexin V-positive cells were mainly located in the right lower quadrant. The apoptotic rate was calculated as the percentage of Annexin V-positive cells divided by the total number of cells. (d) The generation of intracellular ROS was assessed using DCFH-DA. The green signal of DCF was captured by a fluorescence microscope as presented. Scale bar = 50 *μ*m. ROS levels were calculated using the fluorescence value (mean fluorescence intensity (MFI) of DCF) and calibrated with the total RNA concentration. The intracellular ROS levels in different groups are shown in the histograms. Results are presented as means ± SD of *n* = 4‐5 independent experiments. ^∗^Conditions significantly different (*P* < 0.05).

As shown in Figure 2(a), the expression of LEGLTBC in INS-1 cells was significantly decreased by 1.7- and 6.1-fold after treatment with HG/PA for 12 and 24 h, respectively. To prove that LEGLTBC plays roles in glucolipotoxicity-induced islet beta cell injury, the effects of LEGLTBC inhibition on HG/PA-cultured INS-1 cells were investigated. We knocked down LEGLTBC with siRNA (Figure 2(b)) and found that the number of apoptotic cells was markedly enhanced (Figure 2(c)). In addition, LEGLTBC silencing significantly increased ROS production as detected using the DCFH-DA assay (Figure 2(d)).

**Figure 3:**
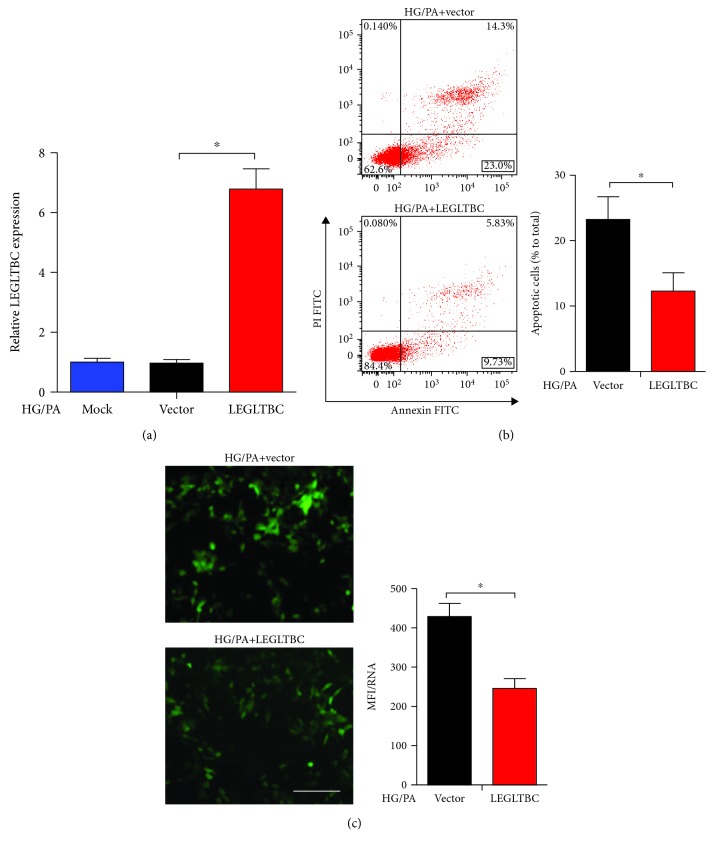
Overexpression of LEGLTBC alleviates oxidative stress and apoptosis in glucolipotoxicity. (a) Enhanced LEGLTBC expression using plasmid for 48 h as detected by qRT-PCR analysis. (b) INS-1 cells were stained with Annexin V-FITC and PI and then subjected to flow cytometry to measure apoptosis. The apoptotic rate was calculated as the percentage of Annexin V-positive cells divided by the total number of cells. (c) The generation of intracellular ROS was assessed using DCFH-DA. The intracellular ROS levels in different groups are shown in the histograms. Results are presented as means ± SD of *n* = 4‐5 independent experiments. ^∗^Conditions significantly different (*P* < 0.05).

To further clarify the biological function of LEGLTBC, we used the pcDNA3.1 vector to construct cell lines overexpressing LEGLTBC (Figure 3(a)). Interestingly, the upregulation of LEGLTBC was shown to reduce apoptosis and ROS and increase the cell growth rate in HG/PA-treated INS-1 cells (Figures 3(b) and 3(c), [Supplementary-material supplementary-material-1]). However, the overexpression of LEGLTBC did not modify the insulin mRNA level and insulin release ([Supplementary-material supplementary-material-1]). These data indicated that LEGLTBC plays a significant role in inhibiting glucolipotoxicity-evoked oxidative stress and apoptosis.

### 3.4. LEGLTBC Functioned as an Endogenous Sponge for miR-34a

**Figure 4:**
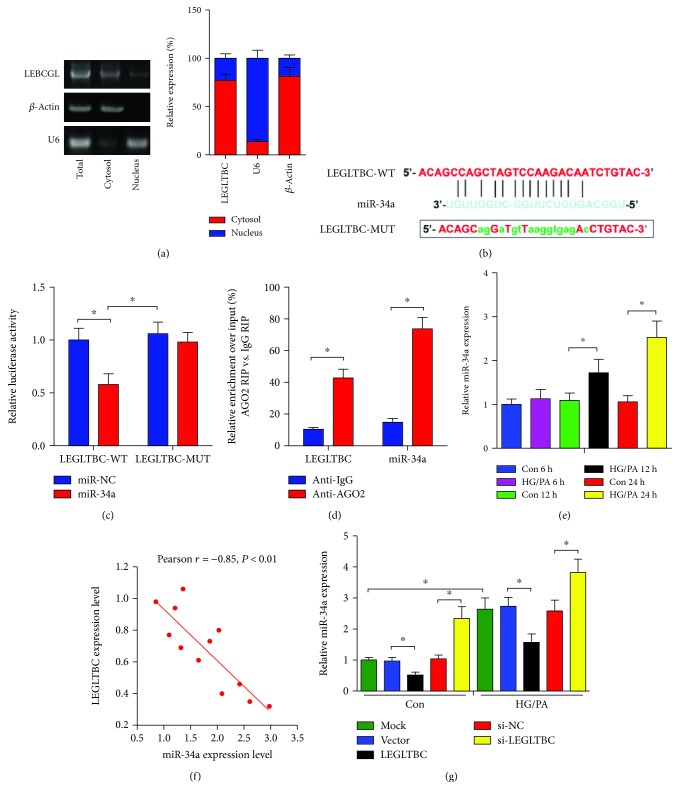
LEGLTBC directly interacts with miR-34a. (a) Levels of LEGLTBC from nuclear and cytoplasmic fractions of INS-1 cells were measured by qRT-PCR, and results showed that LEGLTBC is mainly located in the cytoplasmic fractions. (b) Bioinformatics prediction indicated that the LEGLTBC sequence contains the putative binding site of miR-34a. (c) The luciferase activity was measured in INS-1 cells after having been cotransfected with LEGLTBC-WT or LEGLTBC-MUT and miR-34a or miR-NC for 48 h. (d) The RIP assay was performed to confirm whether LEGLTBC and miR-34a could directly bind to AGO2 in INS-1 cells. (e) The expression of miR-34a in HG/PA-treated INS-1 cells was assessed by qRT-PCR analysis. (f) Bivariate correlation analysis was performed to clarify the relationship between LEGLTBC and miR-34a expression level. (g) qRT-PCR was used to assay miR-34a expressions in INS-1 cells transfected with LEGLTBC, si-LEGLTBC, or respective controls. Results are presented as means ± SD of *n* = 4‐5 independent experiments. ^∗^Conditions significantly different (*P* < 0.05).

Although we found that LEGLTBC could inhibit oxidative stress and apoptosis, how it resulted in these protective effects was unclear. Targeting miRNA is one of the regulatory functions of lncRNA. Given that LEGLTBC contained complementary binding sites to miRNA and located mainly in the cytoplasm fractions (Figure 4(a)), we speculated that LEGLTBC may act as a ceRNA in the biological process. Bioinformatics programs RegRNA (http://regrna2.mbc.nctu.edu.tw) and BiBiServ (https://bibiserv.cebitec.uni-bielefeld.de/rnahybrid) were used to predict the target genes of LEGLTBC. Several miRNAs (miR-34a, miR-412 and miR-199a) were selected for the study according to their relative high affinity between LEGLTBC. These miRNAs were evaluated through qRT-PCR acquired from rat INS-1 cells to investigate the association of LEGLTBC with the expression trend of these three miRNAs. On one hand, the expression trend of miR-412 and miR-199a was not changed in INS-1 cells incubated with HG/PA, which was also unaltered in INS-1 cells transfected with LEGLTBC ([Supplementary-material supplementary-material-1]). On the other hand, miR-34a exhibited an opposing expression trend to LEGLTBC (Figure 4(f)), thereby indicating its potential as a target for LEGLTBC. Thus, miR-34a was selected for further study.

To confirm the direct binding between LEGLTBC and miR-34a, a fragment of LEGLTBC including the putative wild-type or mutated binding sites was constructed into the luciferase reporter vectors (Figure 4(b)). Cotransfection with the miR-34a mimic and LEGLTBC-WT resulted in an obvious decrease of luciferase activity in INS-1 cells, while no significant effect was present in cells transfected with the miR-34a mimic and LEGLTBC-MUT (Figure 4(c)). miRNAs are present in the form of miRNA ribonucleoprotein complexes (miRNPs) which contains AGO2, a core component of the RNA-induced silencing complex [22]. The RIP assay was performed in INS-1 cells to determine whether LEGLTBC was associated with miRNPs. In line with the results of the luciferase reporter assay, LEGLTBC and miR-34a were both specially enriched in the AGO2-containing miRNPs relative to the IgG control (Figure 4(d)).

To clarify the association of LEGLTBC with the expression trend of miR-34a, we assessed the expression of miR-34a at different time points in HG/PA-treated INS-1 cells. As shown in Figures 4(e) and 4(f), miR-34a level was gradually increased along with prolonged incubation in the presence of HG/PA, which exhibited an opposing expression trend to LEGLTBC. To further investigate the regulatory effects of LEGLTBC on miR-34a expression, the LEGLTBC, si-LEGLTBC, or matched controls were transfected into INS-1 cells. As shown in Figure 4(g), overexpression of LEGLTBC obviously downregulated miR-34a expression and LEGLTBC inhibition was found to promote miR-34a expression. These results suggested that LEGLTBC serves as competing endogenous RNAs (ceRNAs) interacting with miR-34a in INS-1 beta cells.

### 3.5. miR-34a Targets SIRT1

**Figure 5:**
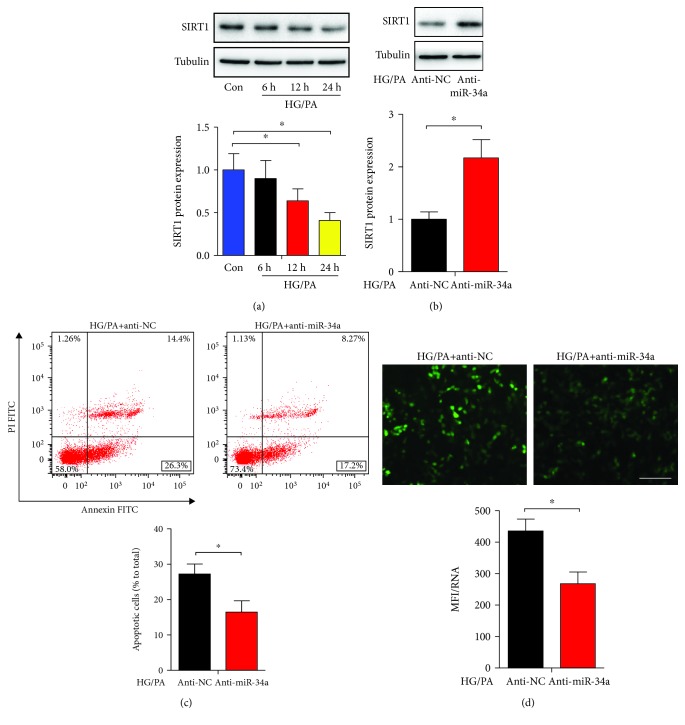
miR-34a targets SIRT1. (a) The expression of SIRT1 in HG/PA-treated INS-1 cells was assessed by western blot analysis. Representative bands of SIRT1 are shown. Histogram represents SIRT1 expression normalized to the corresponding tubulin. (b) Western blot analysis revealed that inhibition of miR-34a increased the SIRT1 expression. (c) INS-1 cells were stained with Annexin V-FITC and PI and then subjected to flow cytometry to measure apoptosis. The apoptotic rate was calculated as the percentage of Annexin V-positive cells divided by the total number of cells. (d) The generation of intracellular ROS was assessed using DCFH-DA. The intracellular ROS levels in different groups are shown in the histograms. Results are presented as means ± SD of *n* = 3‐5 independent experiments. ^∗^Conditions significantly different (*P* < 0.05).

SIRT1 has been demonstrated to be a direct target of miR-34a in the vascular smooth muscle cells [23], retina epithelial cells [24], H9c2 cardiomyocytes [25], mesangial cells [26], and HK-2 cells [27]. Similar with previous studies [13, 28], the incubation of INS-1 cells with HG/PA obviously decreased the protein expression of SIRT1 (Figure 5(a)). Furthermore, the inhibition of miR-34a markedly enhanced the expression level of SIRT1 (Figure 5(b)). Meanwhile, miR-34a depletion reduced the apoptotic INS-1 cells and depressed oxidative stress as illustrated by decreased ROS generation (Figures 5(c) and 5(d)). These data indicated that SIRT1 is the target gene of miR-34a in triggering beta cell apoptosis and ROS generation.

### 3.6. SIRT1 Knockdown Abolished the Protective Effects of LEGLTBC

**Figure 6:**
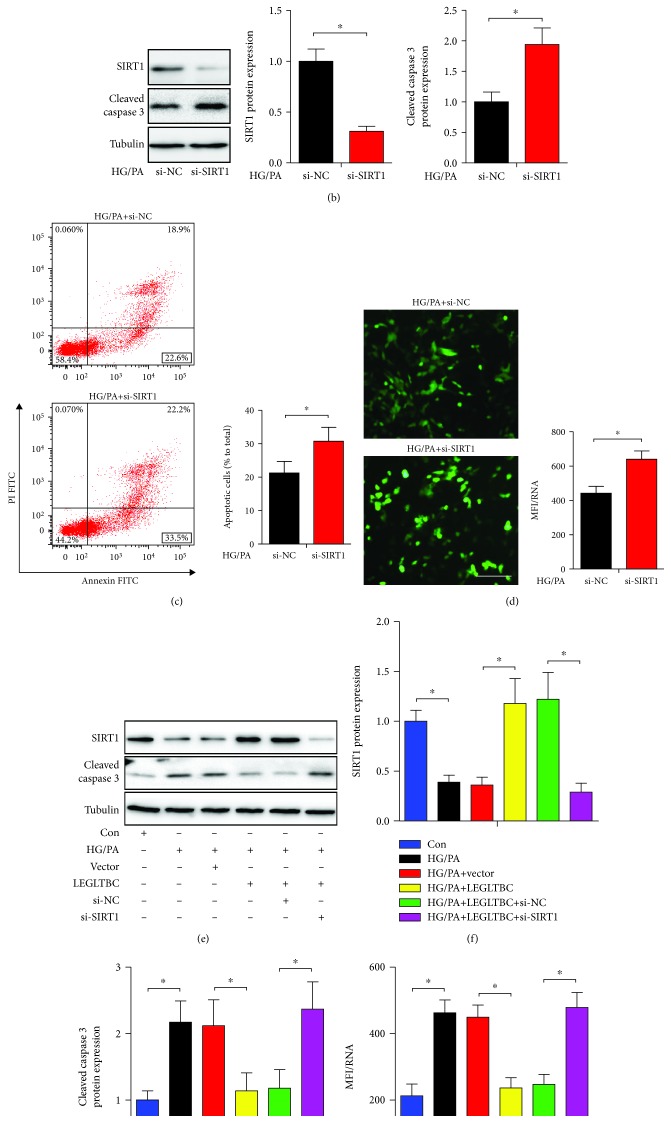
SIRT1 knockdown abolished the protective effects of LEGLTBC. (a) Western blot analysis showed that LEGLTBC silencing or overexpressing positively regulated SIRT1 expression. Knockdown of SIRT1 increased the cleaved caspase 3 expression in HG/PA-treated INS-1 cells (b), which were associated with an obvious increase of apoptotic cells (c) and ROS generation (d). INS-1 cells transfected with vector, LEGLTBC, LEGLTBC+si-NC, and LEGLTBC+si-SIRT1 were treated with HG/PA, followed by the detection of SIRT1 and cleaved caspase 3 expression (e, f, and g) and ROS production (h). Results are presented as means ± SD of *n* = 3‐5 independent experiments. ^∗^Conditions significantly different (*P* < 0.05).

To test whether LEGLTBC regulated the target gene SIRT1 expression through miR-34a, the effects of LEGLTBC on SIRT1 were examined. As shown in Figure 6(a), we found that enforced expression of LEGLTBC dramatically enhanced the SIRT1 expression, whereas silencing LEGLTBC significantly had the opposite effect in HG/PA-cultured INS-1 cells. As mentioned above (Figure 4(g)), LEGLTBC over- or downexpression significantly affected the miR-34a expression. These results suggest that LEGLTBC has positive regulatory effects on SIRT1, the target gene of miR-34a in INS-1 cells.

ROS accumulation is an inducer of pancreatic beta cell apoptosis [29–32]. SIRT1, a nicotinamide adenine dinucleotide-dependent deacetylase, has been demonstrated to alleviate oxidative damage and the associated apoptosis in response to diverse stress conditions [13, 33–35]. Given the important role of SIRT1 in regulating ROS generation, we tested the effects of SIRT1 on glucolipotoxicity-induced INS-1 cell damage. As shown in Figures 6(b), 6(c), and 6(d), SIRT1 knockdown further enhanced the protein expression of cleaved caspase 3 (a key marker of apoptosis), which was associated with an obvious increase of apoptotic cells and ROS production. These data suggested that LEGLTBC protects INS-1 cells against injury induced by glucolipotoxicity via promoting SIRT1.

In order for us to further investigate whether the biological function of LEGLTBC was mediated by regulating SIRT1, INS-1 cells were transfected with LEGLTBC or LGELTBC+si-SIRT1 before HG/PA treatment. As presented in Figures 6(e), 6(f), 6(g), and 6(h), ectopic LEGLTBC expression increased SIRT1 expression in glucolipotoxicity-treated INS-1 cells, while transfection with si-SIRT1 greatly abrogated the LEGLTBC-triggered enhancement of SIRT1 expression. Moreover, the cleaved caspase 3 expression and ROS level in HG/PA-treated INS-1 cells were significantly decreased by upregulating LEGLTBC, whereas the protection by ectopic expression of LEGLTBC was weakened following the introduction of si-SIRT1.

### 3.7. Effects of LEGLTBC Silencing on Rat Pancreatic Islets

**Figure 7:**
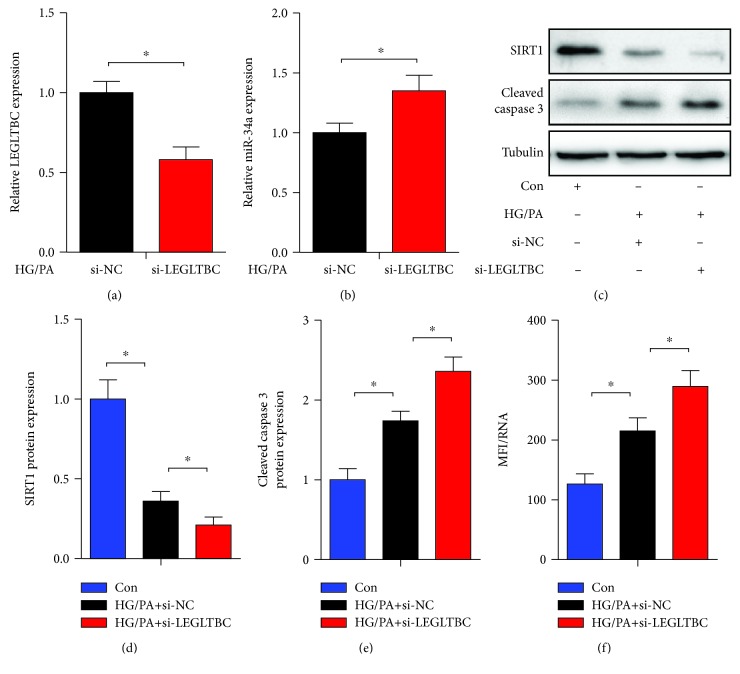
Effects of LEGLTBC silencing on rat pancreatic islets. (a) Downregulation of LEGLTBC using siRNA for 48 h and the expression of LEGLTBC in HG/PA-treated rat islets assessed by qRT-PCR analysis. (b) The expression of miR-34a in HG/PA-treated rat islets was assessed by qRT-PCR analysis. Rat pancreatic islets transfected with si-NC or si-LEGLTBC were treated with HG/PA, followed by the detection of SIRT1 and cleaved caspase 3 expression (c, d, and e) and ROS production (f). Results are presented as means ± SD of *n* = 3‐4 independent experiments. ^∗^Conditions significantly different (*P* < 0.05).

As presented in Figure 7, the knockdown of LEGLTBC significantly upregulated the expression of miR-34a in rat pancreatic islets, which was associated with decreased SIRT1 expression, enhanced cleaved caspase 3 expression, and increased ROS production.

## 4. Discussion

T2DM is the result of a complex interaction between genetic, epigenetic, and environmental factors. Diet excess leading to obesity is considered as the most important factor for T2DM [2]. It has been demonstrated that chronic hyperglycemia and higher levels of circulating FFAs are commonly associated with the beta cell function deterioration [2]. Hyperglycemia could aggravate lipotoxicity-induced beta cell dysfunction (glucolipotoxicity) [5, 6]. Recently, transcriptomics and proteomics have been successfully adopted to discover major changes of protein-coding genes and proteins in pancreatic INS-1 cells exposed to glucolipotoxicity [18, 19]. Here, we revealed for the first time that the incubation of INS-1 cells with HG/PA caused considerable modifications in lncRNA expression.

In the present study, 153 lncRNAs were found to be upregulated, while 111 lncRNAs were downregulated in HG/PA-treated INS-1 cells compared with the control cells using RNA-Seq analysis. Comprehensive analysis shows that lncRNAs are generally expressed at lower levels compared with protein-coding genes [21]. Similar with previous studies [15, 18], we found that the average levels of lncRNA expression in INS-1 cells were lower than those of protein-coding genes. Moreover, some of the differently expressed lncRNAs we identified were randomly selected for qRT-PCR validation in INS-1 cells and rat islets after incubation of HG/PA, and the results confirmed the RNA-Seq results to some extent. These findings may lead towards a better understanding of the function of lncRNAs in the glucolipotoxicity-induced INS-1 beta cell injury.

Moreover, of these differently expressed lncRNAs, we focused on LEGLTBC and demonstrated that HG/PA incubation reduced its expression in INS-1 cells in a time-dependent manner. Enforced expression of LEGLTBC obviously relieved HG/PA-induced beta cell oxidative stress and apoptosis, whereas silencing LEGLTBC significantly had the opposite effects. LncRNAs could act as ceRNAs to regulate the activities and biological functions of mRNAs by competing for binding to shared miRNAs [22]. Herein, using bioinformatics prediction and functional assays, we confirmed that LEGLTBC could directly bind to miR-34a and function as a ceRNA to regulate the expression of SIRT1 in INS-1 cells, which might be a key role involved in HG/PA-induced cell damage.

The evidence for the accumulation of ROS from HG/PA stimulation leading to beta cell dysfunction and apoptosis has been reviewed recently [30–32]. SIRT1 has been demonstrated to play key roles in improving oxidative damage in response to diverse stress conditions [33–35]. Our previous report has demonstrated that miR-199a-5p mediates HG-induced ROS production and apoptosis in INS-1 cells by targeting SIRT1 [13]. Recently, Wang et al. revealed that the inhibition of miR-34a attenuates intestinal ischemia/reperfusion injury through promoting SIRT1-mediated suppression of epithelial ROS accumulation and apoptosis [36]. In the present study, our findings revealed that HG/PA treatment increased the miR-34a expression and downregulation of miR-34a alleviated the glucolipotoxicity-induced beta cell damage by promoting SIRT1. In addition, SIRT1 silencing significantly abolished the protective effects of overexpression of LEGLTBC in HG/PA-cultured beta cells. Collectively, all these data indicated that LEGLTBC functions as a ceRNA and positively regulates the expression of SIRT1 via directly binding to miR-34a. However, it should be noted that miR-34a could regulate a group of target mRNAs other than SIRT1 [10, 12]. Further investigation will be needed to clarify whether these target genes are also regulated by LEGLTBC/miR-34a signaling and contribute to HG/PA-induced beta cell dysfunction.

Although altered lncRNAs were identified and the possible roles of LEGLTBC in HG/PA-induced INS-1 cell oxidative stress and survival were investigated in this study, several limitations should be acknowledged. The RNA-Seq experiments were performed on a small sample size, which may result in an underestimation of the number of altered lncRNAs. Larger sample sizes could achieve more optimal results. In addition, phylogenetic analysis revealed that LEGLTBC is moderately conserved between humans and rats. The reduced expression of LEGLTBC was only observed in rat islets after incubation of HG/PA. Future studies will not only need to elucidate the contribution of LEGLTBC in the development of T2DM in rats but will also need to assess whether the same mechanism operates in humans.

## 5. Conclusions

In the present study, we identified the changes of lncRNA expression in INS-1 cells treated by HG/PA for the first time. Our findings demonstrated that LEGLTBC is downregulated in HG/PA-treated INS-1 beta cells. Mechanistic analysis suggested that the overexpression of LEGLTBC could improve glucolipotoxicity-induced oxidative stress and apoptosis by regulating the miR-34a/SIRT1 axis. Our study provided new insights into the molecular events associated with glucolipotoxicity.

## Figures and Tables

**Table 1:** Sequences of primers.

Primer name	Sequence
NONRATT024184.2 forward	5′-GGTGGAGGGAATGCAAAC-3′
NONRATT024184.2 reverse	5′-CCTTCTCAGCAGTCTGTC-3′
NONRATT006369.2 forward	5′-TCTAAGATAGTGGCTTGATGCT-3′
NONRATT006369.2 reverse	5′-GAAATCAGAGTATGCCCTGAG-3′
NONRATT003679.2 forward	5′-GCTGTACCTCGAATGAATCC-3′
NONRATT003679.2 reverse	5′-AAACCTCTCTCCCAGTAGTC-3′
NONRATT021202.2 forward	5′-GCAGAGGTCTGTTTAGCATAG-3′
NONRATT021202.2 reverse	5′-CTGTGCAGGTCACCTGGTA-3′
NONRATT019034.2 forward	5′-GGAAAGGACCCATATCCCTGA-3′
NONRATT019034.2 reverse	5′-TTATGCCCGGTTCCCAAG-3′
NONRATT009569.2 forward	5′-GTCGTGTCTTCCTTGCTG-3′
NONRATT009569.2 reverse	5′-CTGACTGCAAGAAGTTCTGAT-3′
NONRATT021337.2 forward	5′-GCTCCCAGCTTCACATTG-3′
NONRATT021337.2 reverse	5′-TCCATGCTGGTTGTGACT-3′
NONRATT028292.2 forward	5′-AGTCAGGATGAGGCTTAGT-3′
NONRATT028292.2 reverse	5′-GGTCTGGGTGGAGTTAAGTAG-3′
NONRATT001611.2 forward	5′-CATCTGAGATGGGAGATTGG-3′
NONRATT001611.2 reverse	5′-CCTAAGATAGACACACACAGGA-3′
NONRATT010357.2 forward	5′-AATGTTTACACTAGCCGATCC-3′
NONRATT010357.2 reverse	5′-CACCTTGTCACCTGTTGAG-3′
miR-34a forward	5′-CCAGCTGTGAGTGTTTCTTTG-3′
miR-34a reverse	5′-CAGCACTTCTAGGGCAGTAT-3′
*β*-Actin forward	5′-CCACCATGTACCCAGGCATT-3′
*β*-Actin reverse	5′-CGGACTCATCGTACTCCTGC-3′
U6 forward	5′-CGAGCUGGUAAAGAAUUUATT-3′
U6 reverse	5′-UAAAUUCUUUACCAGCUCGTT-3′
LEGLTBC-WT forward	5′-CCACAGCCAGCTAGTCCAAGACAATCTGTG-3′
LEGLTBC-WT reverse	5′-AACGTGCACTACGAGAACTTTGTT-3′
LEGLTBC-MUT forward	5′-GGTCACAGCAGGATGTTAAGGTGAGACC TGTACCTC-3′
LEGLTBC-MUT reverse	5′-AGCTACATCTGGCTACTGGGTCTC-3′

**Table 2:** Studied lncRNAs with fold changes, *P* values and genomic locations.

LncRNA	Fold change	*P* value	Genomic location
NONRATT015294.2	13.18	5.16 × 10^−7^	Chr2:144866512-144867527
NONRATT010357.2	8.70	4.34 × 10^−11^	Chr15:27230306-27231061
NONRATT003679.2	-5.49	1.28 × 10^−8^	Chr1:245235678-245236805
NONRATT024184.2	-6.00	3.74 × 10^−7^	Chr6:48450847-48658080

## Data Availability

The data used to support the findings of this study are available from the corresponding authors upon request.

## Supplementary Materials

Supplementary Materials The functions of the predicted lncRNA targets were studied via bioinformatics analysis. The DAVID program (http://david.abcc.ncifcrf.gov/) was used to perform GO analysis to identify the molecular functions of potential target genes. Significant correlations between target genes and their associated functions and/or pathways were assessed based on a threshold of *P* < 0.05.
