# Supplementary material for: LncRNA LEGLTBC Functions as a ceRNA to Antagonize the Effects of miR-34a on the Downregulation of SIRT1 in Glucolipotoxicity-Induced INS-1 Beta Cell Oxidative Stress and Apoptosis
Source: Oxid Med Cell Longev. 2019 Oct 14;2019:4010764. doi: 10.1155/2019/4010764 (PMC6815544; doi:10.1155/2019/4010764)
Supplement: Supplementary Materials — The functions of the predicted lncRNA targets were studied via bioinformatics analysis. The DAVID program (http://david.abcc.ncifcrf.gov/) was used to perform GO analysis to identify the molecular functions of potential target genes. Significant correlations between target genes and their associated functions and/or pathways were assessed based on a threshold of P < 0.05. [file 4010764.f1.pdf]

GO analysis The functions of predicted lncRNA targets were studied via bioinformatics analysis. The DAVID program (http://david.abcc.ncifcrf.gov/) was used to perform GO analysis to identify the molecular functions of potential target genes. Significant correlations between target genes and their associated functions and/or pathways were assessed based on a threshold of $p < 0.05$.

In GO enrichment analysis of functional significance, a hypergeometric test was performed for the mapping of all the differentially expressed (DE) mRNAs and DE lncRNAs that target mRNAs to terms in the GO database. In this process, significantly enriched GO terms were search in the DE mRNAs and DE lncRNAs that target mRNAs compared with the genomic background and calculated in accordance with Equation:

$$P = 1 - \sum_{i-1}^{m-1} \frac{\binom{M}{i}\binom{N-M}{n-i}}{\binom{N}{n}}$$

Here, N is the number of all genes with GO annotation, n is the number of DE mRNAs and DE lncRNAs that target mRNAs in N, M is the number of all genes with annotated GO terms and m is the number of DE lncRNAs that target mRNAs in M. The calculated p-value was adjusted through FDR correction, and $p \leq 0.05$ was selected as the threshold. The GO terms that satisfied this condition were defined as significantly enriched GO terms in the DE mRNAs and DE lncRNAs that target mRNAs. The specific pathways significantly enriched in DE mRNAs and lncRNAs with their target genes were then determined.

Determination of cell viability CCK8 (Beyotime) assay was conducted in accordance with the guidelines of the manufacturer to detect cell proliferation. For CCK8 assay, cells were cultivated on 96- well plates for 24 h then incubated in CCK8 for 1 h. The absorbance was measured at 450 nm by the microplate reader.

Measurement of insulin secretion INS-1 cells were starved in Krebs buffer containing 0.2% bovine BSA with 3 mM glucose for 30 min. The cells were then incubated in Krebs buffer with 3 or 25 mM glucose for 45 min. An aliquot of the buffer was taken and insulin release was measured by ELISA.

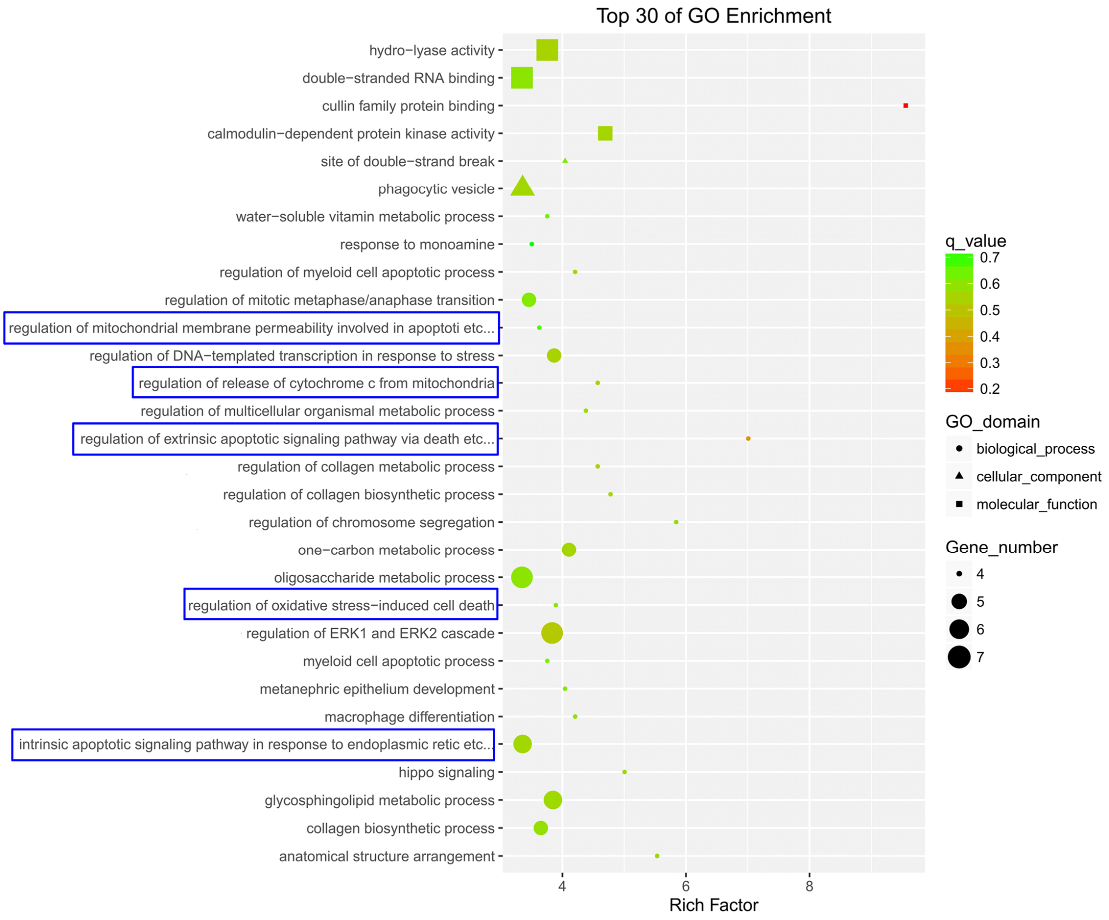

FIGURE S1: GO analysis. The top 30 GO terms that associated with coding gene function of dysregulated lncRNAs are listed. The dysregulated lncRNAs are mainly associated with cell death signaling pathway.

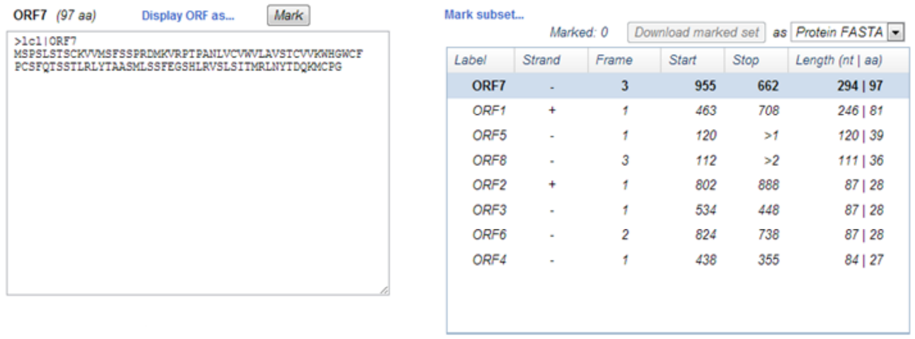

(a)

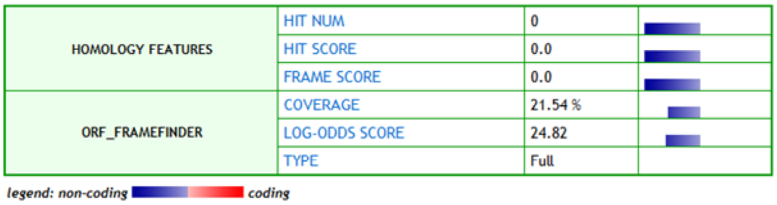

(b)

FIGURE S2: NONRATT003679.2 has no coding capability. (a) Putative proteins possibly encoded by NONRATT003679.2 as predicted by the ORF Finder. (b) The transcript's noncoding nature was suggested by negative score with CPC.

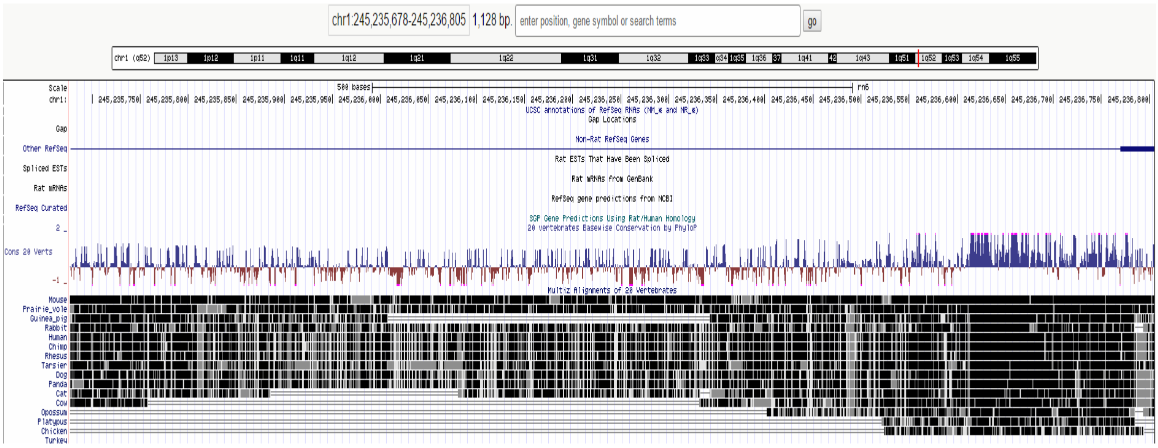

FIGURE S3: Graphical views showing multi-species comparisons of lncRNA LEGLTBC (NONRATT003679.2) using UCSC genome browser. The conservation scores were indicated by the blue and red peaks.

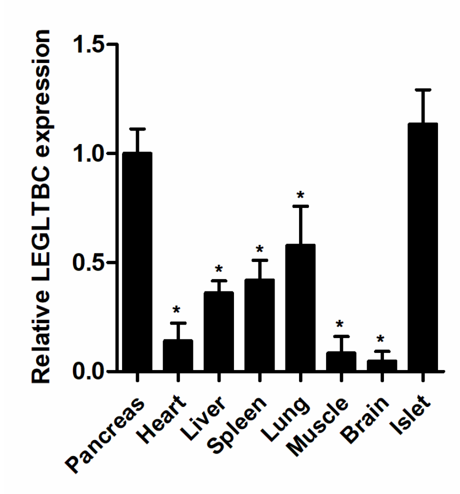

FIGURE S4: qRT-PCR was performed to detect the expression of LEGLBTC in SD rat tissues. The tissues were obtained from 10 week old male SD rats. n = 5. *$P < 0.05$ *VS*. pancreas.

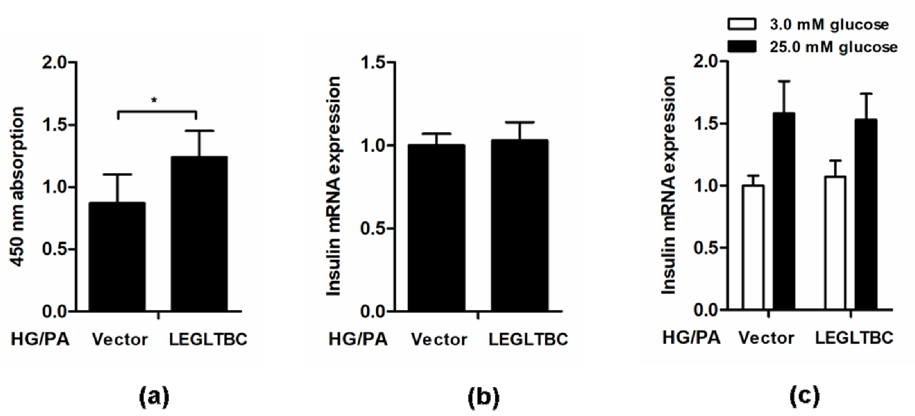

FIGURE S5: Over-expression of LEGLTBC enhances cell proliferation, but does not affect insulin expression or secretion. (a) CCK8 assay was performed to observe the effects on cell proliferation in INS-1 cells after LEGLTBC over-expression. The absorbance was measured at 450 nm by the microplate reader. (b) The expression of insulin mRNA was measured by qRT-PCR. (c) To test the secretory properties, the cells were incubated with 3 or 25 mmol/l glucose for 45 min. Basal and glucose-induced insulin release were assessed by ELISA. Results are presented as means ± SD of n = 5 independent experiments. *Conditions significantly different ($P < 0.05$).

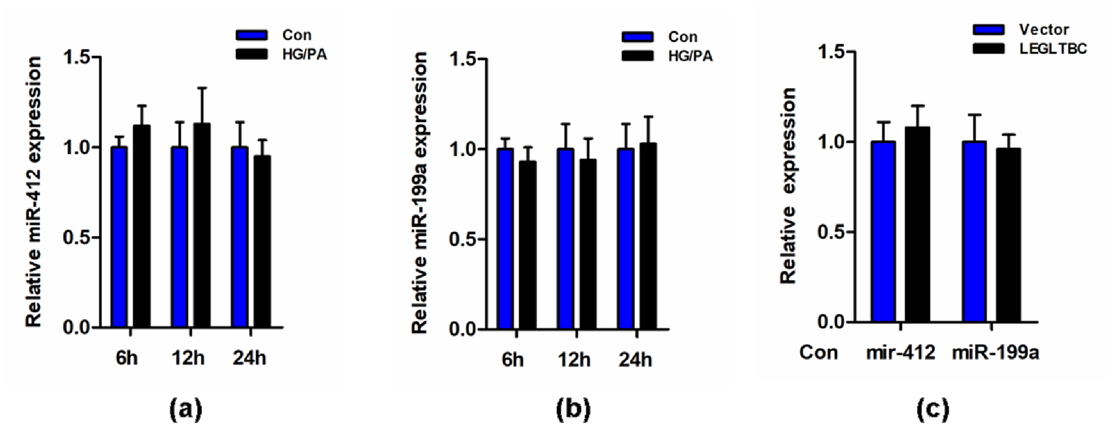

FIGURE S6: The expression trend of miR-412 and miR-199a was not changed in INS-1 cells incubated with HG/PA, which was also unaltered in INS-1 cells transfected with LEGLTBC. The expression of miR-412 (a) and miR-199a (b) in HG/PA-treated INS-1 cells was assessed by qRT-PCR analysis. (c) qRT-PCR was used to assay miR-412 and miR-199a expressions in INS-1 cells transfected with LEGLTBC. Results are presented as means ± SD of n = 5 independent experiments.
